# Body image and sexuality in a sample of 112 of moroccan women with breast cancer

**DOI:** 10.1192/j.eurpsy.2023.785

**Published:** 2023-07-19

**Authors:** S. Elkardi, H. Choujaa, M. Agoub

**Affiliations:** psychiatry, chu ibn rochd, casablanca, Morocco

## Abstract

**Introduction:**

According to the WHO, breast cancer is the number one cancer in women worldwide, and its treatment can have serious effects on the bodies of young women. Surgical treatment can be disfiguring, and chemotherapy can cause an early and abrupt menopause. Each of these treatments can also affect a patient’s sexuality in the short or long term.

**Objectives:**

The aim of our study is to evaluate sexuality and body perception in women with breast cancer after treatment.

**Methods:**

A quantitative descriptive study was carried out among 112 patients followed for breast cancer,majority in sexual activity, met at the consultation of gynecology of the hospital IBN ROCHD Casablanca, Morocco.The data collection was carried out by an information sheet and with the help of two validated scales: BIS (Body image scale) and FSFI (Female sexual function index) in order to evaluate body image and sexuality as well as the HADS (Hospital Anxiety and Depression Scale)

**Results:**

In our sample, 30.6% were older than 50 years, 40.2% were married, 52.2% of the patients came from urban areas, 20.7% of the patients were illiterate, 22.2%had given up their work due to the disease. In terms of family support, 49.4% of the patients were accompanied to the hospital, 52.8% received financial support and 43.8% received moral support.Regarding the relationship of the couple, there is an increase in the frequency of disputes in 49.5%of cases, a change in behavior in 44.9%. The sexual relationship was marked by a decrease in frequency in 36.7%. Concerning the type of treatment received by the 46.8% of the patients had a mastectomy, 20.9% had chemotherapy, 65.5% had radiotherapy and 45.5% had hormone therapy.The prevalence of depression was 54.1%. Its mean score on the HAD scale was 11.46±3.95; that of anxiety was 52.3% with a mean HAD of 11.41±4.04. The prevalence of sexual dysfunction was 100%with a mean FSFI of 14.26±3.68. Body image disturbance was noted in was noted in 83.8% of cases.The factors associated with a body image disorder in the univariate study were marital status (p =0.035; OR = 0.245), educational level (p =0.029; OR = 0.245), depression (p = 0.019; OR = 3.76), and anxiety (p = 0.029;OR = 3.44).

Multivariate analysis of predictors of body image disorder in women with breast cancer

**Table:** 

	**Beta**	**OR ajusté**	**[IC à 95%]**	**P-value**
**education level**	2,229	9,28	[1,89 - 45,60]	0,006
**Marital status**	2,268	9,66	[1,88 – 49,51]	0,007
**Anxiety**	-1,838	0,159	[0,04 – 0,637]	0,009
**Decreased in the quality of sexual relations**	1,368	3,92	[1,08 – 14,17]	0,037
**FSFI scale**	-0,237	0,78	[0,657 – 0,948]	0,011

**Conclusions:**

In total, 4 factors were significantly associated. Given the importance of the subject and the harmful psychological impact on patients further rechearch is needed, also an adequate, emphasized training on the management of women with cancer and their sexual problems and a multidisciplinary work will help improve the psychological state of the women

**Disclosure of Interest:**

None Declared

